# Supplementary figures and images for: Application of video surveillance in preclinical safety studies in canines: Understanding the interobserver reliability and validity to recognize clinical behavior
Source: PLoS One. 2025 Jun 27;20(6):e0326916. doi: 10.1371/journal.pone.0326916 (PMC12204552; doi:10.1371/journal.pone.0326916)

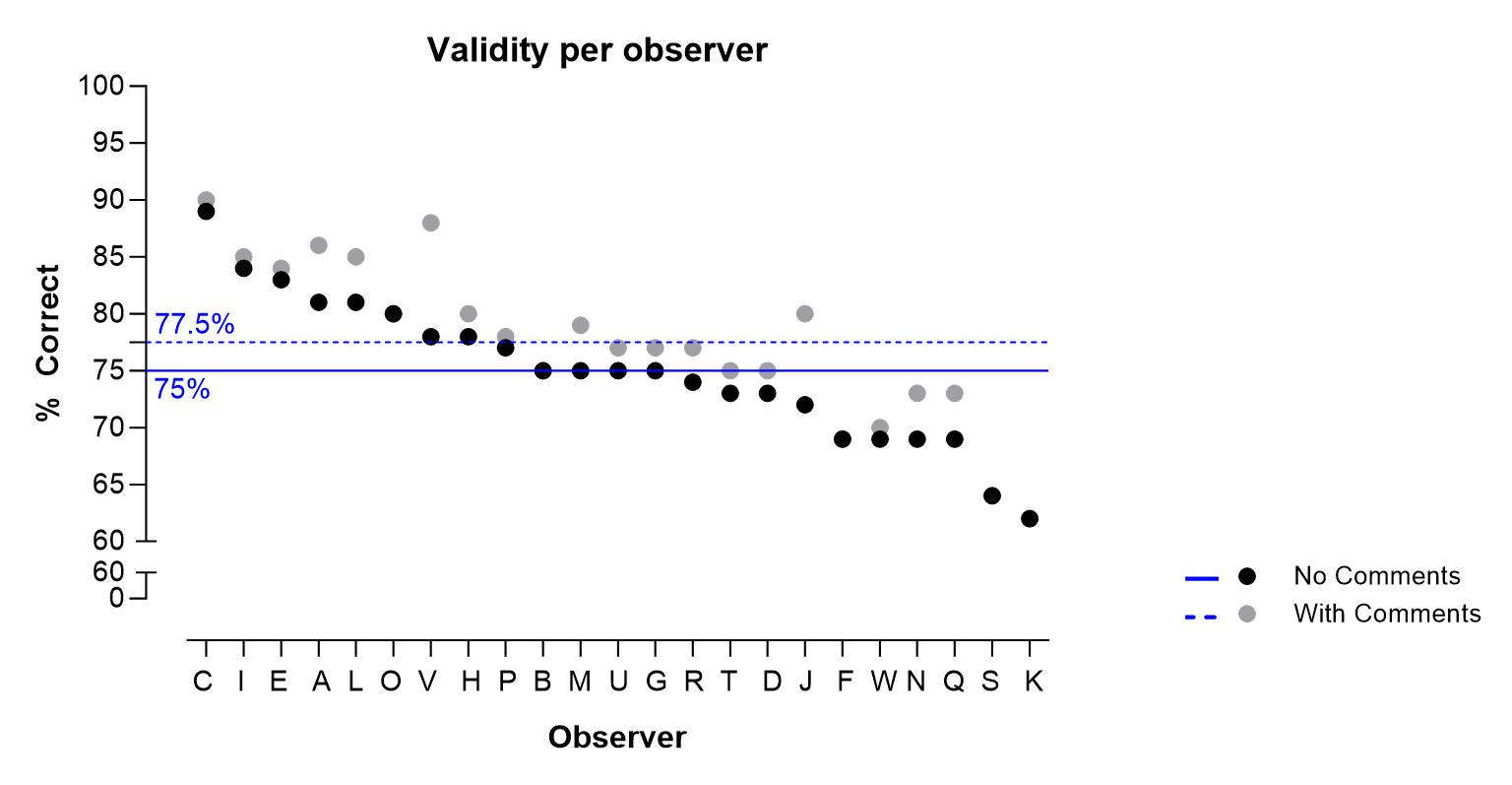

Supplement: S1 Fig — Observers are ranked from high to low validity scores, without (black) and with (grey) comment incorporation. The blue lines represent the overall average validity score across all observers. (TIF) [file pone.0326916.s001.tif]

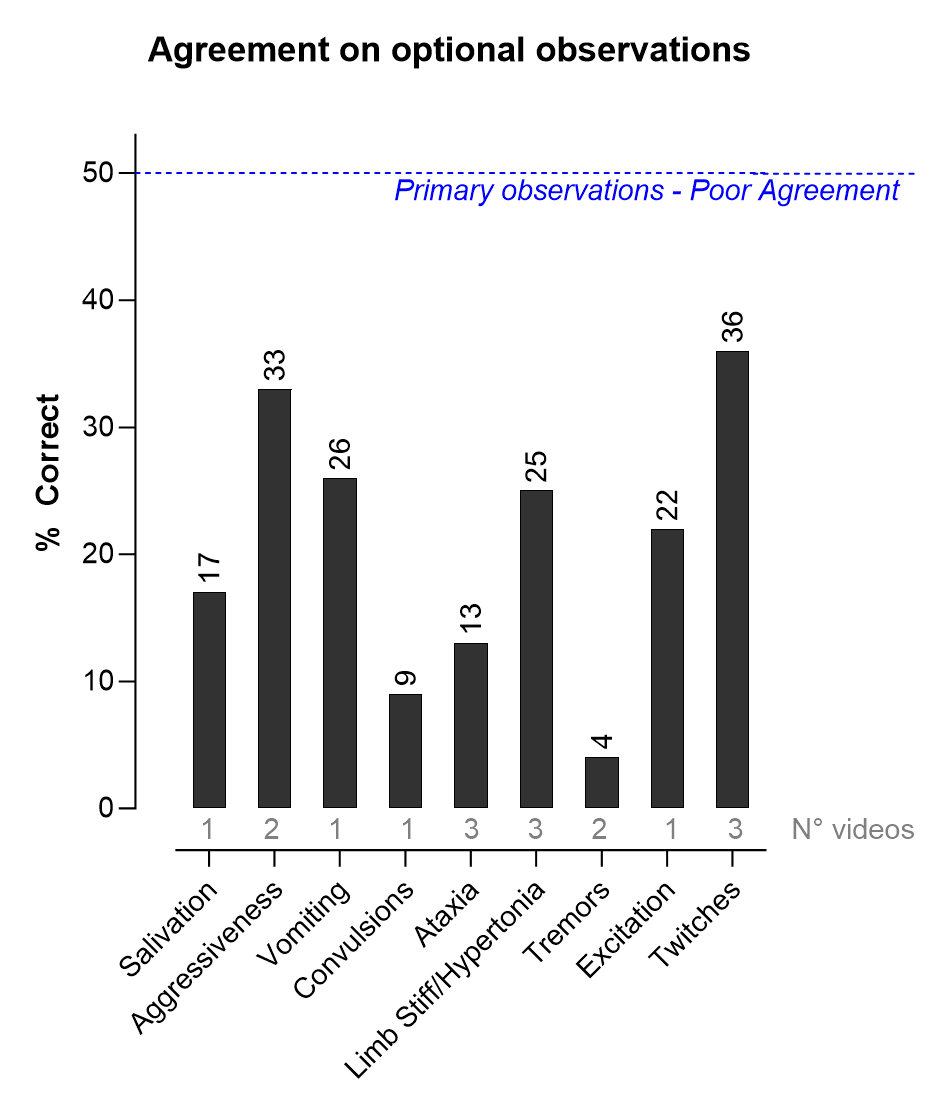

Supplement: S2 Fig — The average % correct score for each optional CO is depicted above each bar; the number of videos containing the respective optional CO is represented in grey below the bars. Data represents results without including comments. (TIF) [file pone.0326916.s002.tif]

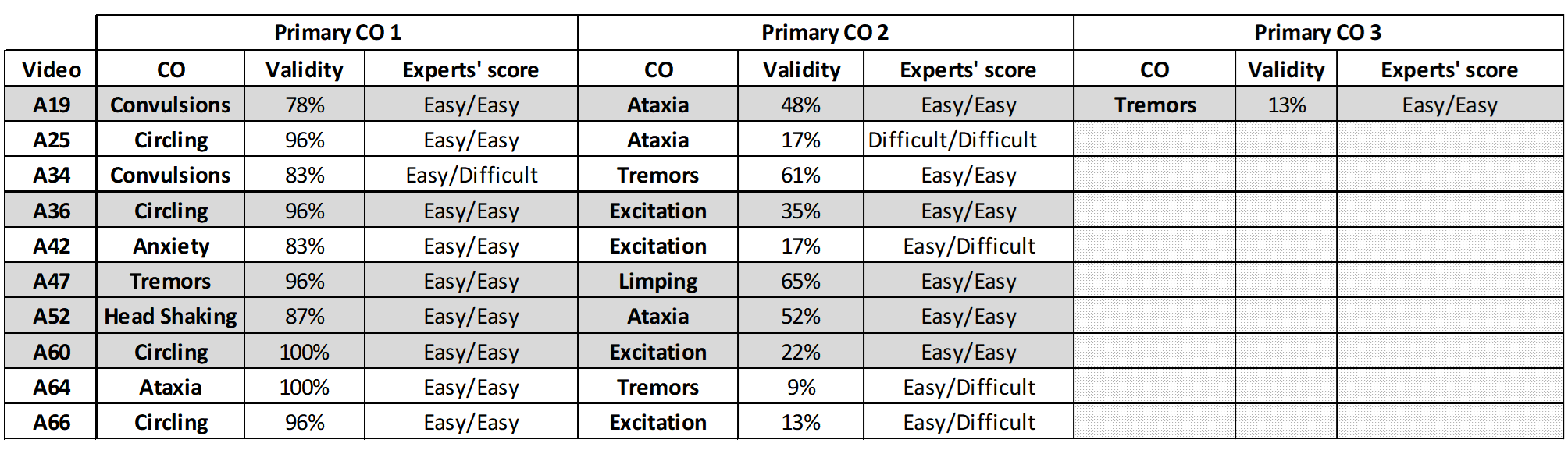

Supplement: S1 Table — For each CO separately in each video, the validity and expert scoring are show. Primary CO 1 reflects the CO with the highest validity, primary COs 2 and 3 reflect the more subtle COs with a lower validity. The highlighted cells (grey) mark the five videos in which all simultaneously occurring COs were marked as easy by both experts. (TIF) [file pone.0326916.s003.tif]

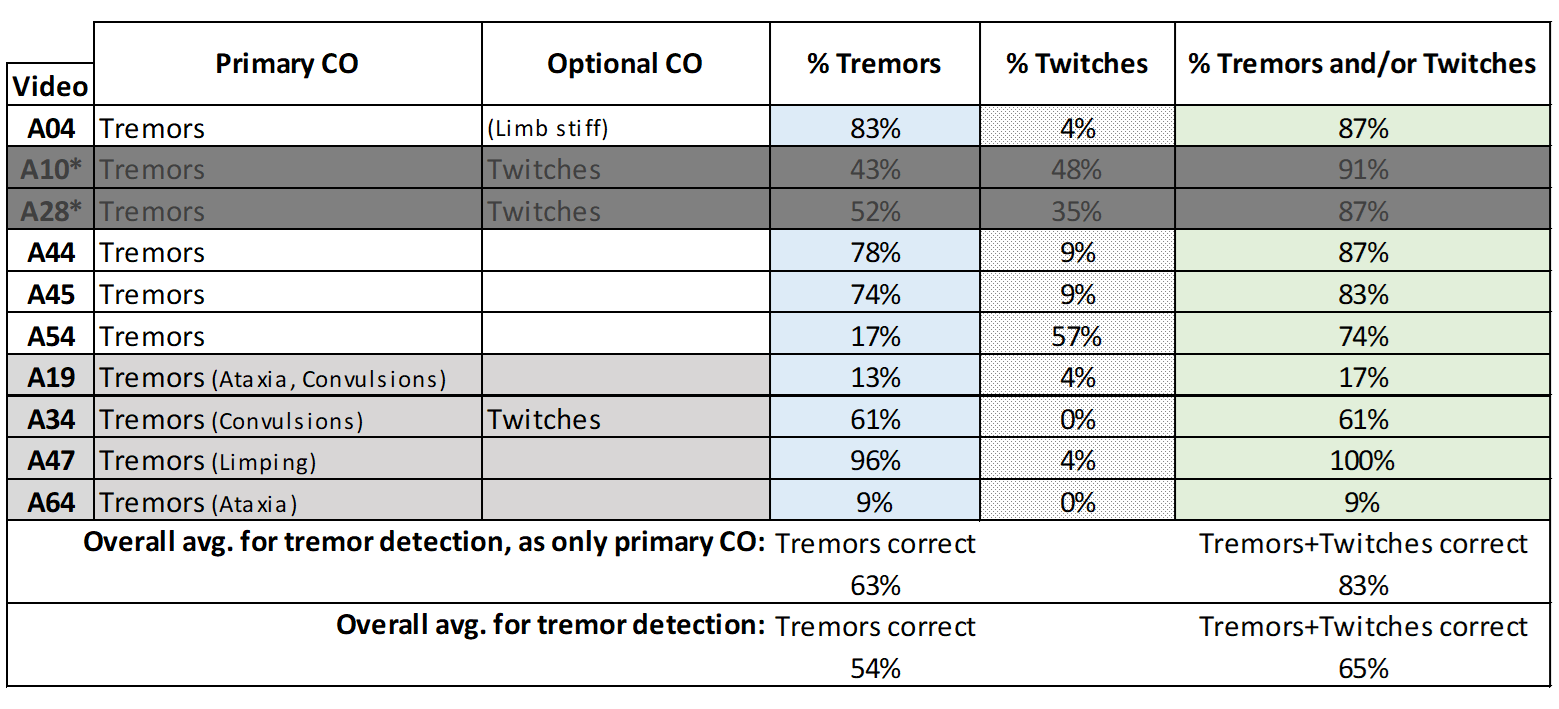

Supplement: S2 Table — Blue values reflect the % of observers that correctly identified the tremors as tremors or as a combination of tremors and twitches, dotted cells are % of observers that scored the tremors as twitches, green values are the % of observers that identified tremors and/or twitches. White cells are the videos in which tremors were present as only primary CO, light grey cells reflect videos in which also other primary COs were present. Videos A10 and A28 were excluded from the analysis as twitches were scored as optional observation by two experts in those videos and they were also detected by a number of observers. (TIF) [file pone.0326916.s004.tif]

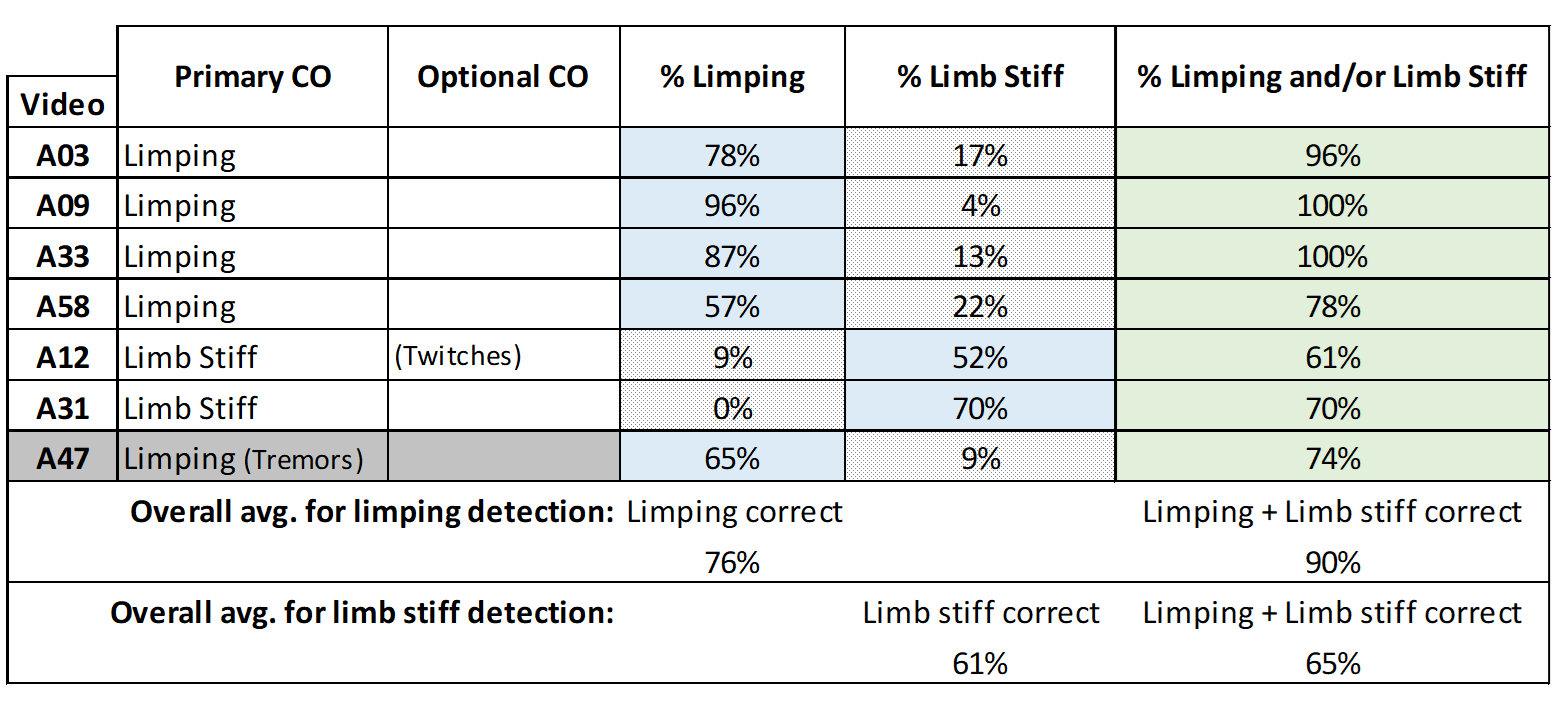

Supplement: S3 Table — Blue values reflect the % of observers that correctly identified limping (four videos) or limb stiff (two videos) or both, dotted cells are % of observers that wrongly scored limping as limb stiff or limb stiff as limping, green values are the % of observers that identified limping and/or limb stiff. White cells are the videos in which limping or limb stiff were present as only primary CO, light grey cells reflect the limping video in which also another primary COs was present. Limping in video A47 was not registered during in-person monitoring. (TIF) [file pone.0326916.s005.tif]

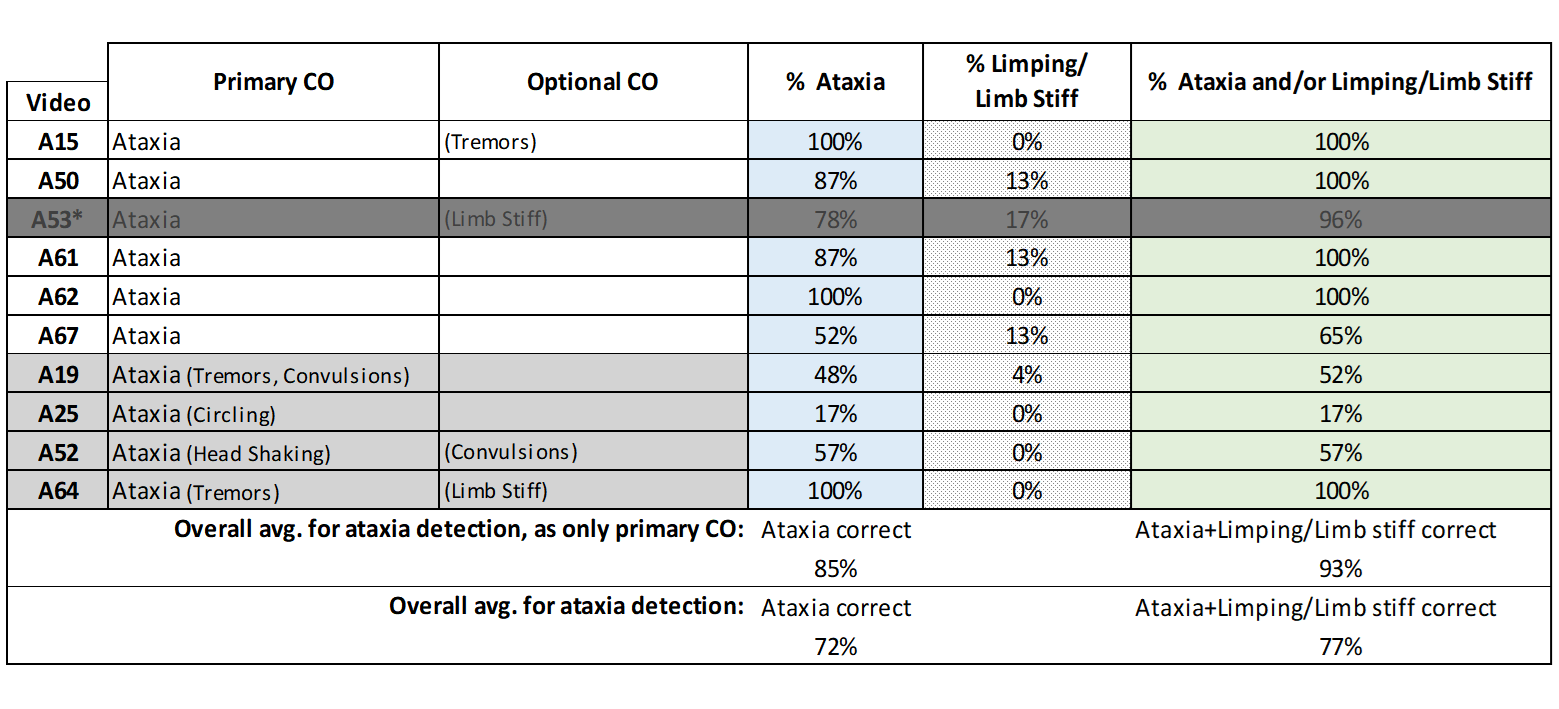

Supplement: S4 Table — Blue values reflect the % of observers that correctly identified ataxia, dotted cells are % of observers that scored ataxia as limping/limb stiff, green values are the % of observers that identified ataxia and/or limping/limb stiff. White cells are the videos in which ataxia was the only primary CO, light grey cells reflect the videos in which also other primary COs were present. Video A53 was excluded from the analysis as limb stiff was scored as optional observation by two experts and it was also identified by several observers. (TIF) [file pone.0326916.s006.tif]
