# Supplementary material for: Application of video surveillance in preclinical safety studies in canines: Understanding the interobserver reliability and validity to recognize clinical behavior
Source: PLoS One. 2025 Jun 27;20(6):e0326916. doi: 10.1371/journal.pone.0326916 (PMC12204552; doi:10.1371/journal.pone.0326916)
Supplement: S1 File — (PDF) [file pone.0326916.s008.pdf]

Results Statistical Analysis

1) Reliability (Interobserver Agreement)

Overall Fleiss's Kappa

Scenario	Fleiss's Kappa
Without comments data	0.70
With comments data	0.72

Fleiss's Kappa for each observation separately

Clinical Observation	Without comments data	With comments data
Salivation	0.99	0.99
Vomiting / Retching	0.95	0.96
Aggressiveness	0.88	0.91
Head shaking	0.87	0.88
Circling	0.81	0.81
Convulsions	0.80	0.82
Ataxia	0.70	0.75
Limping	0.69	0.74
Anxiety	0.69	0.71
Tremors	0.56	0.60
Excitation	0.52	0.53
No Clinical Observation	0.45	0.52
Twitches	0.30	0.29
Limb Stiff / Hypertonia	0.28	0.27
Other Clinical Obs. not listed	0.13	0.07

2) Validity (Observer-Expert Agreement)

Overall Validity

Scenario	Validity
Without comments data	75.0
With comments data	77.5

Validity for each primary observation separately

Primary CO	Without Comments
Salivation	100
Aggressiveness	97
Circling	97
Vomiting / Retching	97
Head shaking	92
Convulsions	90
Limping	77
Anxiety	74
Ataxia	72
Limb Stiff / Hypertonia	61
No Clinical Observation	56
Tremors	53
Excitation	47

Validity per Expertise Level

Expertise	Validity
High	77.0
Moderate	74.3
Minimal	73.3
Avg	74.9

Validity per Primary Role

Primary.Function	Validity
Technician	77.2
Veterinarian	74.3
Scientist	73.7
Avg	75.0

Validity per observer

Observer	Without comments	With comments
C	89	90
I	84	85
E	83	84
A	81	86
L	81	85
O	80	80
V	78	88
H	78	80
P	77	78
B	75	75
M	75	79
U	75	77
G	75	77
R	74	77
T	73	75
D	73	75
J	72	80
F	69	69
W	69	70
N	69	73
Q	69	73
S	64	64
K	62	62

3) **Optional observations**

Agreement on optional observations

Optional CO	Without comments	With comments
Twitches	36	36
Aggressiveness	33	37
Vomiting / Retching	26	30
Limb Stiff / Hypertonia	25	25
Excitation	22	22
Salivation	17	17
Ataxia	13	14
Convulsions	9	9
Tremors	4	4
Overall correct	21.7	22.8

Per Expertise Level (no comments)

Expertise	Validity
High	21.3
Moderate	22.4
Minimal	21.2
Avg	21.6

Per Primary Role (no comments)

Primary.Function	Validity
Technician	22.8
Veterinarian	23.4
Scientist	19.6
Avg	22.0
