# Supplementary material for: Application of video surveillance in preclinical safety studies in canines: Understanding the interobserver reliability and validity to recognize clinical behavior
Source: PLoS One. 2025 Jun 27;20(6):e0326916. doi: 10.1371/journal.pone.0326916 (PMC12204552; doi:10.1371/journal.pone.0326916)
Supplement: Attachment — Submitted filename: Response to Reviewers_E. Eberhardt.docx [file pone.0326916.s010.docx]

**Rebuttal Letter**

*Thank you for your valuable comments. We have addressed all the comments. See our answers in blue italic. Please note that some results were marginally changed upon a final QC check of all data and results.*

1. Please ensure that your manuscript meets PLOS ONE's style requirements, including those for file naming.

*The manuscript, title page and file names have been checked. The reference error codes within the manuscript have been corrected. Everything should be according to the PLOS ONE requirements.*

2. Thank you for stating the following in the Competing Interests section:

“AlI have read the journal's policy and the authors of this manuscript have the following competing interests: all the authors were employed by company Janssen Research and Development, Janssen Pharmaceutical Companies of Johnson & Johnson.”

Please confirm that this does not alter your adherence to all PLOS ONE policies on sharing data and materials, by including the following statement: "This does not alter our adherence to  PLOS ONE policies on sharing data and materials.” (as detailed online in our guide for authors <http://journals.plos.org/plosone/s/competing-interests>).  If there are restrictions on sharing of data and/or materials, please state these. Please note that we cannot proceed with consideration of your article until this information has been declared.

Please include your updated Competing Interests statement in your cover letter; we will change the online submission form on your behalf.

*We confirm that this does not alter our adherence to the PLOS ONE policies. Please find our updated Competing Interests statement in the updated cover letter.*

3. We note that your Data Availability Statement is currently as follows: [All relevant data are within the manuscript and its Supporting Information files.]

Please confirm at this time whether or not your submission contains all raw data required to replicate the results of your study. Authors must share the “minimal data set” for their submission. PLOS defines the minimal data set to consist of the data required to replicate all study findings reported in the article, as well as related metadata and methods (<https://journals.plos.org/plosone/s/data-availability#loc-minimal-data-set-definition>).

For example, authors should submit the following data:

- The values behind the means, standard deviations and other measures reported;

- The values used to build graphs;

- The points extracted from images for analysis.

Authors do not need to submit their entire data set if only a portion of the data was used in the reported study.

If your submission does not contain these data, please either upload them as Supporting Information files or deposit them to a stable, public repository and provide us with the relevant URLs, DOIs, or accession numbers. For a list of recommended repositories, please see <https://journals.plos.org/plosone/s/recommended-repositories>.

If there are ethical or legal restrictions on sharing a de-identified data set, please explain them in detail (e.g., data contain potentially sensitive information, data are owned by a third-party organization, etc.) and who has imposed them (e.g., an ethics committee). Please also provide contact information for a data access committee, ethics committee, or other institutional body to which data requests may be sent. If data are owned by a third party, please indicate how others may request data access.

*We included 2 additional documents under supporting information which contain the minimal data set required to replicate all study findings. 1) An excel file containing the experts’ observations (primary+optional), their scoring and the blinded and completed questionnaires by all 23 observers. 2) A File containing all relevant statistical results. These contain all raw data to replicate the study findings, figures and tables.*

4. We note that Figure 1 in your submission contain copyrighted images. All PLOS content is published under the Creative Commons Attribution License (CC BY 4.0), which means that the manuscript, images, and Supporting Information files will be freely available online, and any third party is permitted to access, download, copy, distribute, and use these materials in any way, even commercially, with proper attribution. For more information, see our copyright guidelines: <http://journals.plos.org/plosone/s/licenses-and-copyright>.

We require you to either (1) present written permission from the copyright holder to publish these figures specifically under the CC BY 4.0 license, or (2) remove the figures from your submission:

a. You may seek permission from the original copyright holder of Figure 1 to publish the content specifically under the CC BY 4.0 license.

We recommend that you contact the original copyright holder with the Content Permission Form (<http://journals.plos.org/plosone/s/file?id=7c09/content-permission-form.pdf>) and the following text:

“I request permission for the open-access journal PLOS ONE to publish XXX under the Creative Commons Attribution License (CCAL) CC BY 4.0 (<http://creativecommons.org/licenses/by/4.0/>). Please be aware that this license allows unrestricted use and distribution, even commercially, by third parties. Please reply and provide explicit written permission to publish XXX under a CC BY license and complete the attached form.”

Please upload the completed Content Permission Form or other proof of granted permissions as an "Other" file with your submission.

In the figure caption of the copyrighted figure, please include the following text: “Reprinted from [ref] under a CC BY license, with permission from [name of publisher], original copyright [original copyright year].”

b. If you are unable to obtain permission from the original copyright holder to publish these figures under the CC BY 4.0 license or if the copyright holder’s requirements are incompatible with the CC BY 4.0 license, please either i) remove the figure or ii) supply a replacement figure that complies with the CC BY 4.0 license. Please check copyright information on all replacement figures and update the figure caption with source information. If applicable, please specify in the figure caption text when a figure is similar but not identical to the original image and is therefore for illustrative purposes only.

*An adjusted replacement figure 1 is supplied that does not contain the copyrighted image.*

Additional Editor Comments :

Please make sure you address reviewer concerns both in your response to reviewers and in the manuscript or indicate why it would inappropriate to do so.


Reviewers' comments:

Reviewer's Responses to Questions

**Comments to the Author**

5. Review Comments to the Author

Please use the space provided to explain your answers to the questions above. You may also include additional comments for the author, including concerns about dual publication, research ethics, or publication ethics. (Please upload your review as an attachment if it exceeds 20,000 characters)

**Reviewer #1:** This manuscript looks at the reliability and validity of using video recordings to assess clinical observations, taking into account observer experience level and role. Overall I think the manuscript is well written and clearly describes the methods and results, while identifying the limitations of video observations, but providing thoughtful suggestions on using such measures to train machine learning technologies for automated detection of behavior. I have a few minor comments/questions below.

Materials and methods:
L127: How were the video snippets selected and by whom?

*We realize that our initial description of this process was indeed unclear. In fact, we have made use of 3 experts from the beginning but only focused on the 2 other experts performing the blinded evaluation in our initial submission (manuscript). Expert 3 selected the video snippets starting from a master list of clinical observations registered on specific timepoints during in-life study evaluations by lab technicians. Expert 3 checked randomly videos matching the timestamp for the specific CO. In case expert 3 confirmed the CO, the video snippet was retained. In general, snippets originated from different studies and/or animals. Experts 1 and 2 then reviewed these videos in a blind fashion and noted their observations. We want to highlight that all three experts come from different groups and have different primary functions and backgrounds – and they do not originate from the same departmental groups.*

*We have adjusted the relevant sections under Materials & Methods in the manuscript to clarify this process (sections 2 and 3).*

L130: What types of studies? Were the animals always in the same housing set up?

*The video snippets are selected from toxicology and mechanistic studies to evaluate non-clinical safety of drug candidates. They originate from 2 different experimental rooms with the same housing set-up. This is now also included under section 2 in Materials & Methods.*

L136: How long were animals separated after feeding?

*Individual housing occurred for a maximum of 4 hours, see now also under section 2 in Materials & Methods*

L140: How were the videos blinded and by whom?

*They were blinded by expert 3 by labeling the video files numerically. The evaluated video snippets did not contain information on the study, animal number, dose, or any other relevant information that might lead to a bias.*

L152-153: Why were twitches only an optional observation?

*Because for all videos containing twitches, only 2 out of 3 experts agreed on the twitches (= our adjusted definition of optional observation following a comment from Revier #2). For primary observations, all 3 experts need to be in agreement, but this was never the case for twitches. This information is now also included in section 3 in Materials & Methods.*

L184: Were the videos randomized in the same order for all observers or randomized per observer? How were videos randomized?

*Videos were randomized by an online tool. They were randomized in the same order for all observers.*

Results:
L273: Error! Reference source not found… - This error is found in numerous places throughout the manuscript.

*All errors are now corrected.*

**Reviewer #2**: The experimental design of the study has some important to note flaws due to the presence of only 2 experts for generating the ground truth for comparison with the observers’ ratings of the videos. Since the ground truth only includes the observations that both experts share, this is essentially sub-selecting only the easier to interpret aspects of the clinical observations and eliminating the role of less easily observed aspects of the animal behavior. This leads the conclusions of the study to be flawed as well since the authors are maintaining that non-experts can interpret as well as experts. However, the authors are only evaluating their correctness based on easier to interpret observations (only those that are shared by 2 experts).

To address these issues, I would recommend major revision of the study design by generating the ground truth based on the observations of more than 2 experts. The ground truth should be observations that show at least a majority prevalence in a sample of exerts to be reflective of the population of exerts instead of only having the study design catered to the observations that overlap between only 2 observers. This automatically eliminates from consideration of the study more difficult to observe behaviors. This leads to the author’s conclusions that the video observations are as accurate as the expert observes overall. If the video observers were assessing behaviors that were more difficult for experts to observe, then it is likely that the accuracy of the video observers would be reduced overall when they are assessing more nuanced behaviors. The current study design introduces potential error if one of the experts makes an error, which could be resolved if more than 2 experts are used to establish the ground truth. The authors should include in their study design justification a power analysis that indicates the rationale for choosing the number of experts given their ability to establish a reliable conclusion. The experts should have a greater inter-expert reliability index than non-experts if they are truly an expert in the subject matter. The study did not conduct this type of analysis for more than 2 subjects to determine what number of experts would contribute to a sufficient baseline.

*We understand the reviewers’ feedback that using only 2 experts poses a potential limitation. We want to clarify that our initial description of the process of video search and selection was unclear and not specified. In fact, we have made use of 3 experts from the beginning but only focused on the 2 other experts performing the blinded evaluation in our initial submission (manuscript). Expert 3 selected the video snippets starting from a master list of clinical observations registered on specific timepoints during in-life study evaluations by lab technicians. Expert 3 checked randomly videos matching the timestamp for the specific CO. In case expert 3 confirmed the CO, the video snippet was retained. In general, snippets originated from different studies and/or animals. Experts 1 and 2 then reviewed these videos in a blind fashion and noted their observations. We want to highlight that all three experts come from different groups and have different primary functions and backgrounds – and they do not originate from the same departmental groups.*

*We have adjusted the relevant sections under Materials & Methods in the manuscript to clarify this process (sections 2 and 3).*

*In our initial manuscript, we did not clearly state that the individual that was selecting the video snippets was an expert as well, but in this revision, it is clearly stated to have opinions of 3 experts. Accordingly, we redefined primary observations (ground truth) as being observed by all 3 observers; and optional observations as being observed by only 2 out of 3 experts. Observations that were only noted by 1 out of 3 experts were discarded. As such, the reviewer’s concern that the current study design introduces potential error if one of the experts makes an error has been addressed. We have adjusted section 3 under Materials & Methods in the manuscript to these updated definitions.*

*Expert 3 initially selected 73 videos, from which 3 videos were rejected since both experts 1 and 2 did not identify the observations in these videos. The updated definition did not change anything on the primary observations in the remaining 70 videos (they were all confirmed by all 3 experts); while 3 optional observations in 3 separate videos were removed as they were only observed by 1 expert (Twitch in video A12, Limb stiff in video A33 and Ataxia in video A47).*

*While we do embrace the feedback of using 3 experts as stated above, we would also like to counter the reviewers’ assumption that only the ‘easier to interpret observations’ remain in our process. Specifically, to include also observations in which no full agreement (2 out of 3) was reached between the experts, we made the distinction between primary and optional observations. The results on the (more difficult) optional observations are also included in the results and discussion section – and are now more elaborate in the revised manuscript.*

*The reviewer also seems to doubt our conclusion that different expertise levels show similar validity. We understand that this might be surprising. To further support our point, we also added the results between expertise levels for the (more difficult) optional observations to the manuscript. These also show that highly experienced observers do not perform better on the optional observations than the moderately or minimally experienced observers. Importantly, we would like to point out that we do see a trend of highly experienced observers missing less actual observations (see results section 6, lines 405-408).*

*This reviewer seems to be very familiar with the very detailed investigation of animal behavior, including behavioral patterns and fingerprinting. We would like to clarify that our clinical observations of interest are related to drug-related effects which in general have obvious visual cues (e.g. vomiting or convulsions). This could explain our results on experts vs non-experts (as is also reported in other studies on more ‘general’ canine behavior, see our discussion). Also, all observers were familiar with canine studies and have received a refresher training, so even the ‘minimally’ experienced group has quite some experience compared to true laymen.*

For example, the authors should reference studies in a range of fields including animal behavior and ethology (Anderson et al 2014; Bohnslav et al 2021) and other fields such as medical image annotation (Athanasiou et al 2023) and radiology (Lebovitz et al 2021), for standards on the number of experts that should be used for establishing the ground truth as these studies use more experts for their study design, ranging from 3 to 23. Since studies in animal ethology have noted that 2 human expert observers only agree around 70%, it would be necessary to obtain a dataset of multiple observers (at least 3-5 experts) so that an observation can be determined to be present in most expert assessments. These standards for study design are especially important given that the authors are making conclusions regarding the relevance of their findings for the area of computational animal behavior, which would require a larger number of experts as a standard for training models than they used in this study.

*Thank you for the very informative references. We are certainly in agreement of using more than 2 experts (see above and updated manuscript). We would like to state that in the referenced articles of the reviewer, a range from 2-4 experts is used to label a dataset. The paper on DeepEthogram for instance states that the majority of its datasets are labeled by 2 experts.*

*We want to clarify that we did not aim to detect detailed behavioral patterns or emotional states, we specifically focused only on drug-related Clinical Observations that are of importance for non-clinical safety studies. We referenced to other studies focusing on more ‘general’ (working) dog behavior, that are more relevant to our setup. The majority of these referenced studies use 2 experts to set the Ground Truth, while some only use a single expert or 3 experts. Nevertheless, in the current revision we have clarified that 3 experts were involved in evaluation of the videos.*

The authors state that their data is not fully available due to restrictions. This does not meet the PLOS One journal requirement for data availability in the current state. The UNESCO Recommendations on Open Science state that restrictions “are only justifiable on the basis of the protection of human rights, national security, confidentiality, the right to privacy and respect for human subjects of study, legal process and public order, the protection of intellectual property rights, personal information, sacred and secret indigenous knowledge, and rare, threatened or endangered species” and that “In cases where data cannot be openly accessible, it is important to develop tools and protocols for pseudonymizing and anonymizing data, as well as systems for mediated access, so that as much data as possible can be shared as appropriate.”. The authors should address these points regarding restriction of their data, and if they are not applicable, then they should provide access to their data in a public repository such as Open Science Framework.

*We included 2 additional documents under supporting information. 1) An excel file containing the experts’ observations (primary+optional), their scoring and the blinded and completed questionnaires by all 23 observers. 2) A file containing all relevant statistical results. These contain all raw data needed to replicate the study findings, figures and tables.*

Overall, if these major concerns are addressed, this would lead to a scientifically rigorous approach as the study goals are worthwhile to examine. As it stands, the issue with using only 2 experts for the study leads to results that are obscured by the elimination of too much data from consideration to be reflective of whether video observers can be as reliable and as valid as experts. The author’s study purpose for evaluating the effectiveness of video analysis is important to extend the ability to acquire data from animal studies, so a more rigorous approach would be important to achieve results that support these goals.

**Reviewer #3**: The current manuscript presents a study assessing the validity and reliability of measures used to evaluate behavioural responses in lab dogs.
The manuscript is well written and clear. However, what authors are describing in this manuscript as original research, is a common part of the standard early steps for measuring behaviour (e.g., ethogram building, observer training and reliability assessment; see Bateson and Martin 2021), with the add on of testing whether level of experience (subjectively and self assessed) has an effect on reliability.
Indeed, the validity and reliability of behavioural measurements in safety assessment studies are quite relevant and needed. Behavioural indicators of clinical concerns in laboratory animals are often missed or unreliably scored; all of which can decrease translatability, the quality of data and compromise the welfare of animals. However, I fail to see how results from the current manuscript contribute to address this issue:

1. The study lacks an independent assessment of the validity of the measures included – video scoring performed by two experienced observers within the same department, does not validate a behavioural/clinical measure. The comparison between experts scoring of videos and the rest of observers is still an indicator of consistency and measures can be reliable/consistent but have low validity – simply put, to which degree a measure is reflective of what it is intended to measure (the phenomenon of interest; e.g., the animal’s clinical or emotional state or the adverse effects of a compound; see Nunnally 1978, for a reading on face and construct validity).

*We want to clarify that our initial description of the process of snippet selection and expert review was not detailed and clear. We have re-written the Materials & Method sections 2 & 3.*

*In fact, we have made use of 3 experts from the beginning but only focused on the 2 other experts performing the blinded evaluation in our initial submission (manuscript).. Expert 3 selected the video snippets starting from a master list of clinical observations registered on specific timepoints during in-life study evaluations by lab technicians. Expert 3 checked randomly videos matching the timestamp for the specific CO. In case expert 3 confirmed the CO, the video snippet was retained. In general, snippets originated from different studies and/or animals. Experts 1 and 2 then reviewed these videos in a blind fashion and noted their observations.*

*We want to highlight that all three experts come from different groups (In Vivo Sciences, Safety Pharmacology and Toxicology) and have different primary functions and backgrounds – and they do not originate from the same departmental groups. We also want to stress that the* in-person *observations (on which basis the video snippets are selected by expert 3) serve as independent assessment of the validity. These* in-person *observations were recorded by trained lab technicians (experience ranging from 5-40y) during the study period.*

*We have adjusted the relevant sections under Materials & Methods in the manuscript to clarify this process (sections 2 and 3).*

2. Definitions of selected clinical observations confound behaviours with the inferences that can be made regarding the animals’ internal emotional and clinical states, and in several cases lack a clear description of objectively measurable behaviours. I understand that these definitions follow an internal glossary, thus are practical and applicable for their research lab, but reduces the relevance of its findings outside their group.

*Clinical observations are a relatively standardized set used in the pharmaceutical industry for non-clinical safety studies. These glossaries are well aligned between companies conducting in-house animal studies and CRO’s performing outsourced animal studies for multiple pharmaceutical companies.*

4. Videos were selected and cut for the purpose of this study. I question whether these videos are representative of the variation (in quality and behavioural diversity) seen in real post dosing cage side observation videos; hence, reliability assessed in these videos may not be translatable to actual video scoring for assessing drug adverse effects.

*All the used video snippets originate from real post dosing cage side observations from historical non-clinical safety studies. To reflect the variation that is observed post-dosing, expert 3 selected snippets of as many different dogs that showed the observation, during as many different studies testing different compounds.*

*It is true that cutting the videos is a limitation – as also stated by multiple observers and included in the discussion. This was however done to limit the observers’ time needed to watch (and re-watch) the 70 videos in a reasonable time frame.*

5. It does not address the issue of how video recordings can be used instead of direct observation.

*We addressed this issue throughout the discussion and conclusion, we apologize if this was not clear. We added a summary paragraph to the discussion for further clarification.*

*In summary, video recordings are a valuable addition to in-person monitoring as they allow to re-watch certain behaviors and COs for more detailed evaluation of the animals; and monitor the animals without human presence. Video recordings can be used instead of direct observation when the latter is not feasible (e.g. outside working hours) for the majority of the clinical observations, keeping in mind that subtle observations or subtle phenotypes of a certain observation might become less clear and can therefore be missed. This could be circumvented by having multiple observers assessing the videos and possibly grouping certain similar observations together, e.g. tremors and twitches.*

Minor: several references marked as: Error! Reference source not found..

*This was indeed an error in the word file, apologies. All errors are now corrected.*

**Reviewer #4**:

1. Rewrite lines 91-92 for better understanding. *Done*
2. Rewrite lines 107-110 for clear understanding. *Done*
3. Could you please explain why there is a huge difference in the time range of the video snippets in the lines 127-128.

*The video length was determined by the length of the observation itself. Some observations are very clear in a short snippet – and these snippets were therefore not made unnecessary longer; while other observations require more context and a longer video snippet for a correct interpretation. In the video with the very long length (6min40), the observation of interest lasted that long, so therefore it was decided not to cut the video.*

4. How many dogs were observed in each video please mention in the line 140.

*There was one animal to be observed in each video. I added this information to line 214. Line 140 talks about the veterinary oversight, so it seemed like the wrong place to include this information.*
