# Supplementary material for: Application of video surveillance in preclinical safety studies in canines: Understanding the interobserver reliability and validity to recognize clinical behavior
Source: PLoS One. 2025 Jun 27;20(6):e0326916. doi: 10.1371/journal.pone.0326916 (PMC12204552; doi:10.1371/journal.pone.0326916)
Supplement: Attachment — Submitted filename: Response to Reviewers 2_E. Eberhardt.docx [file pone.0326916.s011.docx]

**Rebuttal Letter**

*Thank you for your valuable comments. We have addressed all the comments. See our answers in blue italic.*

**Reviewer #2**: The authors have addressed my primary concerns of the number of experts described for establishing the necessary and optional observations for COs as well as the availability of raw data in the manuscript.

There are some remaining minor editorial changes that should be made, including:

Line 161: “When all 2 experts agreed…” should read “When all 3 experts agreed…”

Authors should make sure that they are consistent between the use of numerals and spelled out number notation ie. Three vs 3 and two vs 2 should remain consistent throughout the manuscript. This is an issue throughout the sections of the manuscript, so I will not note each case of inconsistency – the authors should thoroughly review the manuscript to maintain consistent notation. Typically, numbers are spelled out to represent numbers one through nine.

*Thank you for pointing out the inconsistencies. We thoroughly reviewed the manuscript again to correct them. All the numbers are spelled out now, except when referring to expert 1, 2 or 3. These are kept as numerical for clarity.*

**Reviewer #3**: Authors have not yet satisfactorily addressed my first issue - which is non-trivial as it represents part of the core of the manuscript. In fact, this was also brought up by reviewer 1 and your response also did not address his comment. Having 3 instead of 2 experts does not “fix” the issue, their outcome is still a reliability measure rather than an evaluation of validity. Please provide a thorough explanation of the type of validity evaluated.

*We included in the manuscript a thorough explanation of the types of validities that were evaluated (Materials & Methods section 3, Results section 1 and updated Fig 1) and explain further below.*

*Our main purpose was to investigate whether observations made in-life can be correctly recognized on video footage. Therefore, we first evaluated the criterion validity between the in-life observations and experts on video and secondly the construct validity between the experts and observers.*

*For the criterion validity, we investigated whether experts saw the same observations on video as those registered during the in-life phase of the studies. These in-life observations are considered as the current ‘gold standard’ as they are based on an internal lexicon which is well aligned between pharma companies and CRO’s and on which all relevant personnel is trained.*

*To cite Rebecca K Meagher on criterion validity in animal welfare research “to assess something that can be measured directly in some other way by comparing it to an existing ‘gold standard’ measure.” (Rebecca K. Meagher 2009).*

*Out of 103 recorded in-life observations, only five were not observed by ≥2* *experts. This was on two occasions anxiety, on two occasions limb stiff and once twitches. All other 98 observations (81 primary and 17 optional) that were entered in-life were also seen on video by at least two experts. Based on these results, we conclude a high criterion validity of expert ratings on video compared to the ‘gold standard’ in-life observations.*

*For the construct validity of the observer ratings, we compared these to the expert observations (Fig 3 in the manuscript). Since the expert observations have a high criterion validity compared to in-life, they are considered as the ‘best approximate standardized test’ to which we compare the observer ratings.*
